# Suppression of neointima formation by targeting β-catenin/TCF pathway

**DOI:** 10.1042/BSR20160229

**Published:** 2016-12-09

**Authors:** Helen Williams, Sadie Slater, Sarah Jane George

**Affiliations:** * School of Clinical Sciences, Research Floor Level 7, Bristol Royal Infirmary, Upper Maudlin St, Bristol BS2 8HW, U.K.

**Keywords:** β-catenin, gene therapy, neointima formation, restenosis, Wnt

## Abstract

Coronary artery disease is treated by vein grafting and stent implantation. Late vein graft failure and restenosis of stented arteries reduce the success rates of these approaches and are caused by neointima formation. We have previously shown that Wnt proteins are up-regulated during intimal thickening, and have speculated that these lead to activation of downstream genes with β-catenin/T-cell factor (TCF)-responsive promoters. In the present study, we aimed to provide evidence that β-catenin/TCF signalling promotes neointima formation and assess whether targeting this pathway has potential for reducing neointima formation. We utilized a gene therapy approach selectively targeting cells in which the β-catenin/TCF pathway is activated by using a recombinant adenovirus Ad-TOPTK, which carries a herpes simplex virus thymidine kinase (HSV-TK) gene under the control of a β-catenin/TCF-response promoter. Cells with activated β-catenin will therefore be selectively killed. Ad-TOPTK and ganciclovir (GCV) treatment significantly suppressed the growth of the neointima in a murine model of left carotid artery ligation. In summary, we demonstrated that Wnt/β-catenin/TCF signalling promotes neointima formation, by showing that the selective death of cells with activated β-catenin suppressed neointima formation. This highlights the therapeutic potential for reducing late vein graft failure and in-stent restenosis by targeting β-catenin/TCF signalling.

## INTRODUCTION

Migration and proliferation of vascular smooth muscle cells (VSMCs) to form a neointima occur in pathologies such as restenosis after angioplasty and vein grafting. Our recent work has shown the involvement of the Wnt/β-catenin pathway in both migration [1,2] and proliferation [3]. This has been confirmed by work in other groups [4–6]. However, it has never been shown directly that β-catenin/T-cell factor (TCF) signalling is directly required for neointima formation *in vivo*.

We aimed to show that β-catenin/TCF signalling is required for neointima formation and to examine whether using a suicide gene approach, similar to that previously utilized by Kwong et al. [7] in a mouse model of colon cancer, could retard neointima formation following complete carotid artery ligation in mice. To achieve this, we have utilized an adenoviral vector in which expression of the herpes simplex virus thymidine kinase (HSV-TK) gene is driven by a novel β-catenin/TCF-responsive promoter linked to a minimum CMV promoter (Ad-TOPTK). We delivered this adenovirus to the adventitia of the mouse carotid artery after ligation and then implanted a mini-osmotic pump to infuse ganciclovir (GCV). If the carotid ligation and induction of neointima formation resulted in increased β-catenin signalling, this would lead to production of thymidine kinase (TK) by the TOPTK virus. Expression of TK would in turn convert the inactive prodrug GCV (9-[2-hydroxy-1-(hydroxyethoxy)ethoxy]methylguanine; GCV) into toxic GCV triphosphate resulting in the selective death of cells with β-catenin activity, and thereby reducing neointima formation.

In summary, the present study will not only test directly whether β-catenin/TCF signalling is required for neointima formation but also be a proof of principle study to demonstrate whether a β-catenin /TCF signalling driven suicide gene approach retards neointima formation. If this is the case, future approaches could be developed to utilize this strategy to reduce vein graft failure and in-stent restenosis.

## MATERIALS AND METHODS

### Adenoviruses

Ad-TK and Ad-TOPTK viruses were generously provided by Mien-Chie Hung (Department of Molecular and Cellular Oncology, MD Anderson Cancer Center, The University of Texas, Houston, Texas). These viruses have been described in detail previously [7]. Production of Ad-β-galactosidase (Ad-β-gal) [8] and Ad-m-β-cat (encoding constitutively active β-catenin) have been described previously [9].

### Human VSMC culture and infection

Surplus de-identified segments of human saphenous vein were collected at the completion of bypass surgery from patients that gave written informed consent for the use of their tissues for research. The use of these vein segments for the present study was approved by NHS National Research Ethics Service (NRES number 04/Q2007/6: Frenchay Ethics Research Committee). It was not necessary for the Faculty of Health Sciences Ethics Board, University of Bristol to approve the present study as it was approved by NRES. However, the University of Bristol Research Governance approved, registered and sponsored the present study. Human saphenous vein smooth muscle cells (SMCs) were isolated from the saphenous vein segments using the explant technique [10] and maintained in Dulbecco's Modified Eagle's medium supplemented with 10% (v/v) FBS and antibiotics. Cells were utilized between passages two and six. Cells were infected at ∼70% confluency. An accurate cell count was performed prior to infection. Cells were infected with 1000 plaque forming units (pfu)/cell of Ad-TK, Ad-TOPTK or Ad-β-gal with or without 300 pfu/cell Ad-m-β-cat or Ad-β-gal for 18 h in complete media. Subsequently, cells were washed with PBS and cultured for a further 64 h in fresh serum-free media in the presence or absence of 10 μg/ml GCV. To quantify cell number, cells were analysed using the water-soluble tetrazolium (WST) assay. In brief, WST-1 reagent (Roche Molecular Biochemicals) was added to cells following the manufacturer's instructions, incubated at 37°C for 2 h and then the absorbance at 450 nm measured to quantify cell number.

### Husbandry

C57bl6/J mice were obtained and housed in the animal facilities at the University of Bristol. Housing, care and all procedures were performed in accordance with the guidelines and regulations of the University of Bristol and the United Kingdom Home Office. The investigation conforms to the Guide for the Care and Use of Laboratory Animals published by the US National Institutes of Health (NIH Publication No. 85-23, revised 1996). The University of Bristol, Animal Welfare and Ethical Review Body (AWERB) approved the present study. The left common carotid artery was ligated near the carotid bifurcation, as described previously [3], to induce neointima formation. Following the ligation, the artery was surrounded by 30% (w/v) pluronic gel containing 0.44 × 10^10^ pfu Ad-β-gal, Ad-TK or Ad-TOPTK adenovirus. An osmotic mini-pump (Catalogue number 2002 and 2004, Alzet) was inserted subcutaneously that released GCV at 25 mg/kg per day or PBS as control.

### Histochemistry and immunohistochemistry

The ligated carotid arteries were removed 3 and 21 days later and embedded in agar blocks vertically or horizontally respectively for sectioning. Agar blocks were processed and embedded in paraffin wax. Sections were cut at 3 μm thickness and stained using elastin Van Gieson (EVG) for visualization and quantification of the vessel and neointima by image analysis (Image Pro). The size of the intima was measured and then expressed as a percentage of the lumen area. Immunofluorescence staining for TK was performed using 4 μg/ml goat anti-HSV-1 TK (Santa Cruz Biotechnology: VN-20 SC-20837) to show expression of the virus following adenoviral infection and ligation. Briefly, sections were deparaffinized, rehydrated and subjected to antigen retrieval using citrate buffer and microwaving, sections were blocked with Image-FX and then incubated with the primary antibody overnight. The following day the bound primary antibody was detected with anti-goat Alexa Fluor-488 and then mounted with prolong gold with DAPI. Non-immune goat IgG was used as negative control.

### Western blotting

Carotid arteries were removed, rinsed in PBS, chopped finely and lysed in 5% (w/v) SDS lysis buffer with agitation for 15 min, before centrifugation at 13.000 g for 5 minutes and removal of supernatant for Western blotting using 50 ng/ml goat anti-HSV-1 TK (Santa Cruz Biotechnology: VN-20 SC-20837). Bound primary antibodies were detected using HRP-conjugated secondary antibodies and ECL reagent (GE Healthcare). Protein loading was observed using Bio-Rad stain-free technology.

### Statistics

Results are expressed as mean ± S.E.M. Data were analysed by ANOVA and Student–Newman–Keuls post hoc test when more than three groups were analysed and Student's *t* test when two groups were analysed. A significant difference accepted when *P*<0.05.

## RESULTS

### Selective killing of VSMC with β-catenin signalling

**Figure 1:**
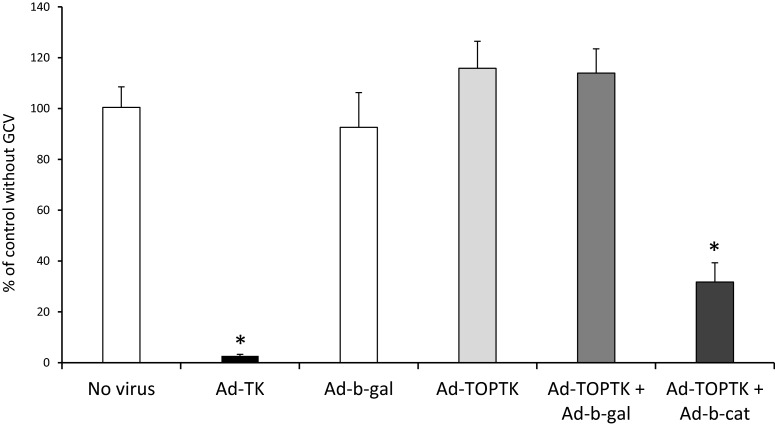
VSMCs with increased levels of active β-catenin are selectively killed by Ad-TOPTK Human saphenous vein VSMCs were infected with 1000 pfu/cell Ad-TK, Ad-β-gal, Ad-TOPTK with or without 300 pfu/cell Ad-β-gal or Ad-β-catenin and cultured in the presence or absence of GCV. Cell number was measured by WST-1 and the % of control without GCV was calculated. *n*=3, * indicates significant difference from no virus, Ad-β-gal, Ad-TOPTK and Ad-TOPTK  + Ad-β-gal, ANOVA and Student–Newman–Keuls post hoc test.

In order to test the hypothesis that VSMCs can be selectively killed by targeting β-catenin/TCF pathway with a responsive promoter, VSMCs were infected with Ad-TOPTK with or without Ad-β-catenin and cultured in the presence or absence of GCV. Cell number was measured by WST-1 assay, to assess cell viability. The efficacy of Ad-TOPTK in cell killing was compared with the adenovirus with TK under the control of the complete CMV promoter (Ad-TK). As shown in Figure 1, almost all cells subjected to infection with Ad-TK and cultured in the presence of GCV were killed. Infection with Ad-β-gal, Ad-TOPTK or Ad-TOPTK  + Ad-β-gal and culture in the presence of GCV had no significant effect on cell death (Figure 1). In contrast, cell death was significantly increased in VSMCs infected with Ad-TOPTK + Ad-β-catenin compared with uninfected cells and VSMCs infected with Ad−β-gal, Ad-TOPTK or Ad-TOPTK + Ad-β-gal (Figure 1). This result indicated that in the presence of GCV, the adenoviral vector Ad-TOPTK could selectively induce death in VSMCs with enhanced β-catenin signalling. These results indicated that Ad-TOPTK plus GCV treatment could be used in gene therapy to selectively kill VSMC without affecting VSMCs basal levels of β-catenin signalling.

### Infection of carotid artery with adenoviruses

**Figure 2:**
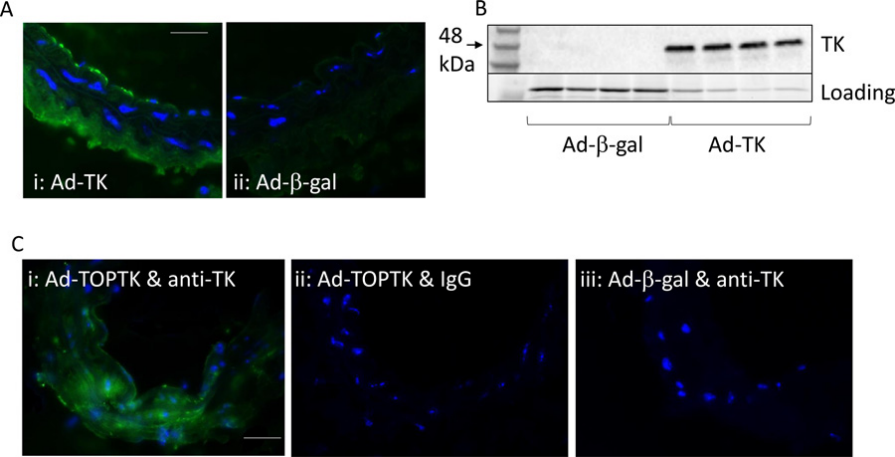
Expression of TK in the carotid artery after application of Ad-TK and Ad-TOPTK Ligated carotid arteries (*n*=4 per group) were coated with pluronic gel containing Ad-TK or Ad-β-gal as control. After 3 days, the arteries were subjected to either immunofluorescence for TK (**A**) or Western blotting for TK (**B**). Ligated carotid arteries (*n*=4 per group) were coated with pluronic gel containing Ad-TOP-TK or Ad-β-gal as control. After 3 days, the arteries were subjected to immunofluorescence for TK (**C**). TK is stained green and nuclei are stained blue with DAPI in immunofluorescence (A and C). Negative control arteries infected with Ad-TOPTK were subjected to immunofluorescence using non-immune immunoglobulin (C). Scale bar represents 20 μm and applies to both IHC panels.

The effectiveness of infecting the carotid artery of mice with adenoviruses using pluronic gel was examined using the Ad-TK and Ad-TOPTK virus; Ad-β-gal was used as a negative control. The virus was applied to the adventitia immediately after ligation in pluronic gel. The animal was allowed to recover and 3 days later the carotid arteries were removed. Arteries were either fixed in formalin and subjected to immunofluorescence for TK or chopped finely and lysed in 5% SDS lysis buffer. Figure 2 illustrates that TK protein was detected by both immunofluorescence (Figure 2A) and Western blotting (Figure 2B) in the ligated artery, whereas it was undetectable in arteries infected with Ad-β-gal.

### Activation of β-catenin after carotid ligation

To demonstrate activation of the β-catenin signalling pathway after carotid ligation, Ad-TOPTK was delivered to the adventitia of ligated carotid arteries using pluronic gel. Ad-β-gal was used as a negative control. Figure 2(C) illustrates that following application of Ad-TOPTK virus, TK protein was detected in the ligated artery, whereas it was undetectable in arteries infected with Ad-β-gal. This shows that β-catenin/TCF activity in the vessel following carotid ligation was sufficient to activate expression of TK via the TOP promoter.

### Suppression of neointima formation by Ad-TOPTK/GCV

**Figure 3:**
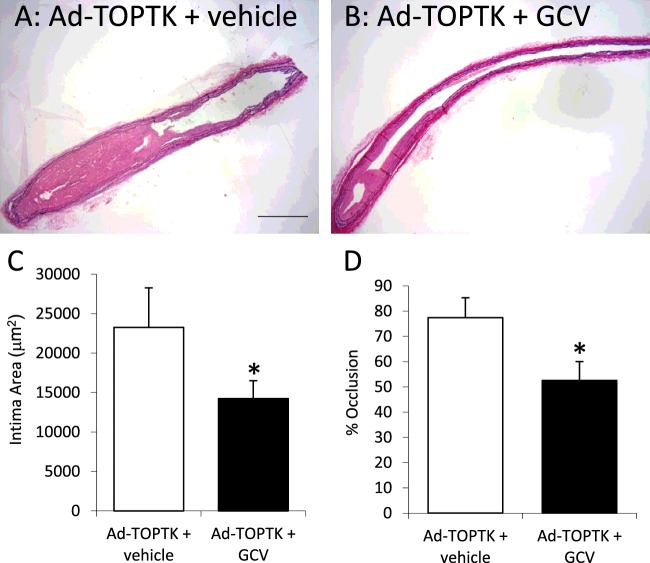
Effect of Ad-TOPTK/GCV on neointima formation Representative examples of EVG stained sections of carotid arteries infected with Ad-TOPTK without GCV treatment (**A**: *n*=8) and Ad-TOPTK with GCV treatment (**B**: *n*=10). Scale bar represents 200 μm and applies to both panels. Intimal area (**C**) and percentage of occlusion of the artery (**D**) were quantified. *indicates *P*<0.05 compared with control without GCV, Student's *t* test.

To test the effectiveness of Ad-TOPTK/GCV in suppressing neointima formation in animals, the adenovirus was delivered to the adventitia of ligated carotid arteries using pluronic gel. In addition, these animals received mini-osmotic pumps to continuously deliver GCV for 21 days and the size of the neointima was measured from paraffin wax embedded sections. As shown in Figure 3, Ad-TOPTK virus with GCV treatment suppressed neointima formation and occlusion of the carotid artery. These results were consistent with the hypothesis that suicide gene expression driven by the β-catenin/TCF-sensitive TOP promoter can effectively suppress neointima formation. GCV alone did not affect intima size or occlusion (19905±3856 compared with 23255±5328 μm^2^ and 63±12 compared with 77±8% respectively).

## DISCUSSION

The present study shows that selective death of cells with activated β-catenin can reduce neointima formation *in vivo*. Although previous studies have implied the involvement of this pathway using reporter animals, detection of β-catenin activity and up-regulation of β-catenin target genes [3,4,11]; direct evidence demonstrating a causal role of β-catenin/TCF signalling in neointima formation has not been reported. It was important to gather this evidence as β-catenin activity alone does not conclusively demonstrate a causal role in disease, as previously noted by Marchand et al. [12]. Furthermore, identification of a causal role highlights the possibility of future therapeutic approaches aimed at targeting this pathway. This strategy could be potentially utilized to retard intimal thickening in vein grafts and stented arteries and thereby reduce failure rates of these two techniques.

When embarking on the present study, we considered the β-catenin/TCF signalling pathway as a very promising approach due to the expanding evidence that was suggestive of a role for β-catenin/TCF signalling in neointima formation. β-catenin is usually sequestered at the cell membrane by N-cadherin, where it is a component of the cadherin cell–cell contacts. During cell migration and proliferation, we have shown that the cell–cell contacts are lost, N-cadherin is shed from the cell extracellular membrane by proteases including matrix degrading metalloproteinases, and β-catenin is released into the cell [10,13]. This free β-catenin is then able to act as a signalling molecule, so in the presence of Wnt proteins it can lead to activation of TCF/LEF and therefore changes in gene expression including up-regulation of cyclin D1 and down-regulation of p21 [1–3]. Moreover, our group has shown *in vivo*, using TOPgal transgenic mice that  β-catenin activity is increased in the media of left carotid arteries 3 days after carotid ligation injury and in the neointima formed 28 days after injury [9].

The present study uses a targeted cell death approach, which has been successfully utilized by groups working in cancer biology [7,14–16]. It is well established that β-catenin/TCF signalling plays a role in cancer cell proliferation [14], and consequently targeting this pathway has been previously utilized to suppress tumour growth [7,15,16], but this approach has not previously been applied to a cardiovascular disease model. Kwong et al. [7] used the same approach as utilized in our study to show that colon cancer was suppressed in mice given Ad-TOPTK/GCV. Similarly, suppressed colon cancer growth was observed in mice treated with adenovirus overexpressing the apoptosis inducer p53-up-regulated modulator of apoptosis (PUMA) under the control of the TOP promoter [15]. Although Chen and McCormick [16] demonstrated a similar gene therapy strategy targeting colon cancer by a β-catenin/TCF-response promoter driving the expression of the pro-apoptotic gene *Fadd*. Beneficial effects have also been observed by inhibition of proliferation of pancreatic and gastric cancer cells using the β-catenin promoter to drive the expression of suicide genes [17,18].

Although it is unlikely to be feasible to directly translate this approach to humans due to the requirement for continual GCV infusion, we consider that this proof of principle study was critical to provide the foundation to examine other suicide gene approaches for the use in restenosis and late vein graft failure. Accordingly, future approaches should avoid the requirement for GCV in order to be useful therapeutics. In addition, it is important that the suicide gene chosen for future work is not secreted, to ensure containment of the cell death to only cells with β-catenin/TCF signalling and not within non-activated cells in the close vicinity. Further work is needed to investigate the effect this approach has on endothelial cell function since this is an important mediator of neointima formation. Wnt/β-catenin signalling is known to affect endothelial cell migration and proliferation [19], so it is essential to demonstrate that targeting this pathway is not detrimental to endothelial cell survival or function, for it to be a useful therapy. It is well established that other signalling pathways such as Erk/Ras/Mek also regulate neointima formation [19], but our study illustrates that selective induction of cell death in cells through activation of a suicide gene by β-catenin/TCF signalling is sufficient to attenuate neointima formation.

**Figure 4:**
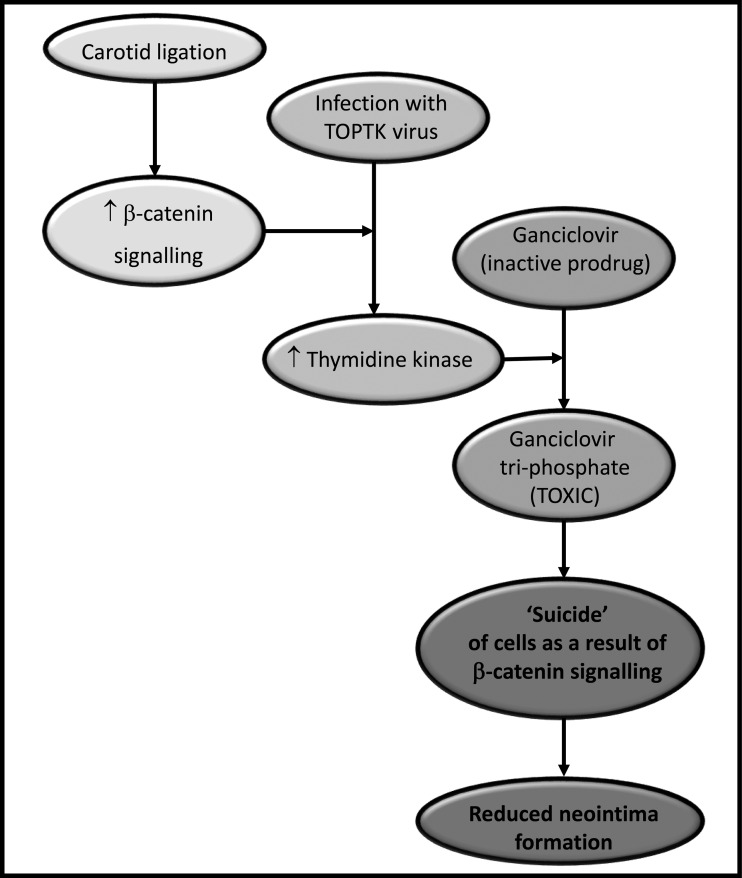
Schematic representation of the reduction in neointima formation by Ad-TOPTK adenovirus and GCV treatment Carotid ligation leads to activation of β-catenin/TCF signalling in the presence of Ad-TOPTK and GCV, this leads to the production of TK that converts GCV into toxic GCV-triphosphate. This leads to ‘suicide’ of cells and reduction in neointima formation as a result of β-catenin/TCF signalling.

In conclusion, we have demonstrated that β-catenin/TCF signalling was activated within the neointima of ligated arteries. We also showed that the combination of Ad-TOPTK adenovirus and GCV treatment selectively killed β-catenin-activated cells, both in tissue culture and in an *in vivo* animal model (summarized in Figure 4). Thus, our work demonstrates that this approach may have therapeutic potential for the reduction in neointima formation and thereby may attenuate the failure rate of vein grafts and stented arteries. In the long term, this may improve the success rates of treatments used for patients with coronary artery disease.

## Abbreviations

Ad-β-gal: Ad-β-galactosidase
Ad-TK: Ad-thymidine kinase
CMU: cytomegalovirus
ErK: extracellular signal-regulated kinases
EVG: elastin Van Gieson
GCV: ganciclovir
HRP: horseradish peroxidase
HSV-TK: herpes simplex virus thymidine kinase
LEF: lymphoid enhancer binding factor
MeK: mitogen-activated
NRES: National Research Ethics Service
pfu: plaque forming units
SMC: smooth muscle cell
TCF: T-cell factor
TK: thymidine kinase
VSMC: vascular smooth muscle cell
WST-1: water-soluble tetrazolium salts-1
